# Predominant role of active versus facilitative glucose transport for glucagon-like peptide-1 secretion

**DOI:** 10.1007/s00125-012-2585-2

**Published:** 2012-05-26

**Authors:** H. E. Parker, A. Adriaenssens, G. Rogers, P. Richards, H. Koepsell, F. Reimann, F. M. Gribble

**Affiliations:** 1 Cambridge Institute for Medical Research, Wellcome Trust/MRC Building, Addenbrooke’s Hospital, Box 139, Hills Road, Cambridge, CB2 0XY UK; 2 Institute of Anatomy and Cell Biology, University of Würzburg, Würzburg, Germany

**Keywords:** Glucagon-like peptide-1 (GLP-1), Glucokinase, K_ATP_ channel, L cells, SGLT1

## Abstract

**Aims/hypothesis:**

Several glucose-sensing pathways have been implicated in glucose-triggered secretion of glucagon-like peptide-1 (GLP-1) from intestinal L cells. One involves glucose metabolism and closure of ATP-sensitive K^+^ channels, and another exploits the electrogenic nature of Na^+^-coupled glucose transporters (SGLTs). This study aimed to elucidate the role of these distinct mechanisms in glucose-stimulated GLP-1 secretion.

**Methods:**

Glucose uptake into L cells (either GLUTag cells or cells in primary cultures, using a new transgenic mouse model combining proglucagon promoter-driven *Cre* recombinase with a ROSA26tdRFP reporter) was monitored with the FLII_12_Pglu-700μδ6 glucose sensor. Effects of pharmacological and genetic interference with SGLT1 or facilitative glucose transport (GLUT) on intracellular glucose accumulation and metabolism (measured by NAD(P)H autofluorescence), cytosolic Ca^2+^ (monitored with Fura2) and GLP-1 secretion (assayed by ELISA) were assessed.

**Results:**

L cell glucose uptake was dominated by GLUT-mediated transport, being abolished by phloretin but not phloridzin. NAD(P)H autofluorescence was glucose dependent and enhanced by a glucokinase activator. In GLUTag cells, but not primary L cells, phloretin partially impaired glucose-dependent secretion, and suppressed an amplifying effect of glucose under depolarising high K^+^ conditions. The key importance of SGLT1 in GLUTag and primary cells was evident from the impairment of secretion by phloridzin or *Sglt1* knockdown and failure of glucose to trigger cytosolic Ca^2+^ elevation in primary L cells from *Sglt1* knockout mice.

**Conclusions/interpretation:**

SGLT1 acts as the luminal glucose sensor in L cells, but intracellular glucose concentrations are largely determined by GLUT activity. Although L cell glucose metabolism depends partially on glucokinase activity, this plays only a minor role in glucose-stimulated GLP-1 secretion.

**Electronic supplementary material:**

The online version of this article (doi:10.1007/s00125-012-2585-2) contains peer-reviewed but unedited supplementary material, which is available to authorised users.

## Introduction

Glucagon-like peptide-1 (GLP-1) is an incretin hormone secreted from intestinal L cells, located throughout the gut epithelium particularly in the ileum and colon [1]. GLP-1 augments insulin secretion in a glucose-dependent manner, and, together with glucose-dependent insulinotropic polypeptide (GIP), is responsible for up to 70% of the insulin response to food intake in healthy individuals [2, 3]. As GLP-1 additionally inhibits glucagon secretion, slows gastric emptying and enhances satiety, it is an attractive target for pharmaceutical interventions. Recent therapies that increase GLP-1 activity, by the use of degradation-resistant mimetics or inhibition of GLP-1 cleavage by dipeptidyl peptidase IV, improve glycaemia in patients with type 2 diabetes, demonstrating the success of targeting the GLP-1 axis. Current interest in studying pathways underlying GLP-1 release could lead to new therapeutic strategies for increasing endogenous GLP-1 secretion.

GLP-1 release is triggered by ingestion of carbohydrates, fats and protein, and is believed to reflect, at least in part, the direct sensing of luminal nutrients via the apical processes of L cells. The release of GLP-1 and GIP after glucose ingestion accounts for the incretin effect, characterised by enhanced insulin release triggered by oral compared with intravenous glucose [4, 5]. This difference can most easily be explained by a ‘luminal’ glucose sensor, shielded from variations in the plasma glucose concentration. The identity of this ‘sensor’ for detecting luminal sugars has been much debated but not fully elucidated.

Initial studies using the GLUTag cell line suggested that L cells may use the classical glucose-sensing machinery used by the pancreatic beta cell, involving glucose phosphorylation by glucokinase, enhanced glycolytic and mitochondrial metabolism, and closure of ATP-sensitive potassium (K_ATP_) channels [6]. More recent studies confirmed that the K_ATP_ channel subunits, *Kir6.2* and *SUR1*, and *Glucokinase* are expressed at high levels in purified mouse L cells and that the proteins are detectable by immunostaining in human L cells [7–9]. Electrophysiological and secretion studies have demonstrated that K_ATP_ channels are functional in murine L cells and that sulfonylureas can stimulate GLP-1 secretion from primary colonic cultures [7].

An element of glucose sensing by L cells is, however, clearly independent of metabolism, as GLP-1 secretion is also stimulated by non-metabolisable sugars such as methyl-α-glucopyranoside (αMG) and 3-*O*-methylglucose in whole animals [10], intestinal preparations [11], GLUTag cells and primary intestinal cultures [7, 12]. Early in vivo studies showed that luminal sugar stimulation of GLP-1 secretion is Na^+^ dependent and that the specificity of the response paralleled the sugar specificity of Na^+^-coupled glucose transport [11, 13]. This led to the identification of a distinct glucose-sensing pathway in L cells, resulting from the activity of Na^+^-coupled glucose transporters (SGLTs) [12]. These concomitantly carry Na^+^ ions for each glucose molecule transported, thereby generating small depolarising currents sufficient to trigger electrical activity and Ca^2+^ entry and consequent increased GLP-1 secretion.

As the relative importance of SGLTs, intracellular glucose levels and metabolism for glucose-dependent GLP-1 secretion is unclear, we aimed to further investigate the roles of these mechanisms in determining L cell glucose fluxes and GLP-1 release.

## Methods

### Animal models

Animal procedures were approved by the local ethics committee and conformed with UK Home Office regulations. *Sglt1* ^*−/−*^ mice [14] on a C57BL/6 background were crossed with GLU-Venus transgenic mice [7]. *Sglt1* ^*−/−*^ and *Sglt1* ^*+/+*^ littermates received a glucose/galactose-reduced diet (Altromin, Lage, Germany).

Labelling of intestinal L cells with a red fluorescent protein (RFP) was achieved by crossing Rosa26tdRFP reporter mice [15] with mice expressing *Cre* recombinase under the control of the proglucagon promoter (GLU-Cre12 mice). GLU-Cre12 mice were created using a construct based on the bacterial artificial chromosome (BAC) RP23-343C17 (Children’s Hospital Oakland Research Institute, Oakland, CA, USA) in which the sequence between the proglucagon start codon in exon 2 and the stop codon in exon 6 was replaced by *iCre* using Red/ET recombination technology (Genebridges, Heidelberg, Germany) (see electronic supplementary material [ESM Methods/Table 1] for more details).

### Tissue culture

Intestines from 3- to 6-month-old mice were collected, and the epithelial cells cultured as described previously [7]. The upper (∼top third) small intestine (SI) comprised a 10 cm length distal to the stomach, and the colon was taken distal to the ileocolic junction. Aliquots were plated on to 24-well plates or 35 mm glass-bottomed dishes (MatTek, Ashland, MA, USA) coated with Matrigel (BD Biosciences, Oxford, UK) for 24–48 h and incubated at 37°C in 5% CO_2_. GLUTag cells were cultured as described previously [16, 17].

### Intracellular glucose measurements

The FLII_12_Pglu-700μδ6 Förster resonance energy transfer (FRET) glucose sensor [18] was cloned into pShuttle-CMV (Qbiogene, Carlsbad, CA, USA) for generation of adenoviruses [19]. GLUTag cells were transfected with pcDNA3.1 containing the glucose sensor under cytomegalovirus (CMV) promoter control (Addgene, Cambridge, MA, USA), using Lipofectamine 2000 (Invitrogen, Paisley, UK). Cells were then seeded on to Matrigel-coated glass-bottomed dishes and imaged 24–48 h later. Before imaging, GLUTag cells were incubated in saline buffer (see below) for 10 min at room temperature. Two-day-old primary colonic cultures from GLU-Cre-×−tdRFP mice were transduced with adenovirus encoding the FLII_12_Pglu-700μδ6 glucose sensor and imaged 72 h later. Before each experiment, colonic cultures were incubated in forskolin (10 μmol/l) and 3-isobutyl-1-methylxanthine (100 μmol/l) for 30 min at 37°C. L cells were identified by their RFP fluorescence and characteristic morphology. FRET imaging was performed using an inverted fluorescence microscope (Nikon Eclipse TE2000-S or Olympus IX71) with a × 40 oil immersion objective. The FRET probe was excited every 5 s at 434/10 nm using a 75 W xenon arc lamp and monochromator (Cairn Research, Faversham, UK) controlled by MetaFluor software (Molecular Devices, Wokingham, UK). Emission was recorded with a CCD camera (QuantEM Photometrics, Tucson, AZ, USA or Orca-ER, Hamamatsu Photonics, Welwyn Garden City, UK) behind an Optosplit II image splitter (Cairn Research) equipped with cyan fluorescent protein (CFP) and yellow fluorescent protein (YFP) emission filter sets. Fluorescence was recorded from individual cells, background corrected and expressed as the ratio YFP/CFP. For analysis, data were averaged over 15–20 s, and peak responses normalised by dividing by the pretreatment baseline.

### Immunohistochemistry

Tissues were fixed with 4% (*w*/*v*) paraformaldehyde for 24 h, cryoprotected in 30% sucrose and embedded in Optimal Cutting Temperature compound (OCT; CellPath, Newton, UK). Tissue sections (8 μm) were permeabilised with 0.05% Tween, blocked with 3% BSA for 1 h, and incubated with 1:300 diluted glucagon antibody (catalogue no. sc-13091, Santa Cruz Biotechnology, Santa Cruz, CA, USA) or 1:50 diluted Glut2 antibody (catalogue no. sc-7580; Santa Cruz) overnight at room temperature. Tissues were then incubated for 1 h at room temperature with Alexa 488-conjugated goat anti-rabbit antibody or donkey anti-goat Alexa Fluor 633 (1:300 dilution; Invitrogen) and Hoechst stain. Tissue samples stained with secondary antibody alone served as controls. Images were captured using either an inverted fluorescence (Olympus IX71) or confocal (Zeiss LSM510) microscope and processed using Volocity and MetaFluor software.

### Assay of glucokinase activity

Glucokinase activity was determined using an adapted pyridine nucleotide-coupled assay [20]. Briefly, ∼3 × 10^7^ GLUTag cells were homogenised in buffer containing (in mmol/l) 25 Hepes, 150 KCl, 2 dithiothreitol and 1 EDTA (pH 7.4), and the resultant supernatant fraction was mixed with assay buffer containing (in mmol/l) 100 Hepes, 6 MgCl_2_, 5 ATP, 0.1% BSA, 150 KCl, 1 dithiothreitol, 1 β-NAD, 0.045 5-thio-d-glucose 6-phosphate, 1 3-*O*-methyl-*N*-acetylglucosamine (Axxora, Exeter, UK), 2.5 IU/ml glucose-6-phosphate dehydrogenase from *Leuconostoc mesenteroides* and glucose (0–60 mmol/l) in a 96-well plate. NADH fluorescence (excitation 320 nm, emission 460 nm) was measured every 1 min for 2 h at 30°C using a Fluoroskan Ascent plate reader (Thermo Scientific, Basingstoke, UK).

### NAD(P)H imaging in GLUTag cells

NAD(P)H levels were imaged in GLUTag cells cultured for 24–48 h on Matrigel-coated glass-bottomed dishes. Cells were washed with and incubated in saline buffer for ∼15 min, mounted on an Olympus IX71 microscope with ×40 oil-immersion objective, and imaged using an Orca-ER CCD camera and Metafluor software. Cells were perfused with saline buffer (plus glucose as indicated) at room temperature. Autofluorescence at 360/15 nm excitation, 510/80 nm emission, was measured every 10 s, background corrected, and averaged over 60 s periods. Mean fluorescence under test conditions was normalised by dividing by the mean of baselines measured before addition, and after washout, of the test reagent.

### Analysis of primary L cells by flow cytometry

Single-cell digests were fixed with 4% paraformaldehyde in PBS for 30 min at room temperature and blocked with PBS/10% goat serum at 4°C overnight. Cells were permeabilised with 0.1% vol./vol. Triton X-100 in PBS/10% goat serum for 30 min at room temperature, and then incubated with or without primary antibody detecting proglucagon (source as above; 1:200 dilution) in PBS/10% goat serum at room temperature for 3 h. Cells were rinsed three times in PBS/10% goat serum and incubated for 1 h with secondary antibody (Alexa Fluor 488; Invitrogen, Eugene, OR, USA; catalogue no. A-11034 at 1:300 dilution). After three washes with PBS, cells were analysed using a BD LSRFortessa analyser (BD Biosciences, San Jose, CA, USA) equipped with 488 nm and 561 nm lasers for excitation of Alexa Fluor 488 and RFP, respectively. Data were analysed using FlowJo 7.6 software (Tree Star, Ashland OR, USA).

For NAD(P)H measurements from primary L cells, SI tissue from GLU-Venus mice was digested to single cells [7] and analysed on the same day. Cells were washed and resuspended in saline buffer containing 0.1% BSA and various glucose concentrations (0–30 mmol/l) 10–15 min before analysis. From each condition, 10^6^ events were assessed using an LSRFortessa analyser and FlowJo 7.6 software. Venus-positive L cells were selected on the basis of their characteristic fluorescence when excited at 488 nm, and autofluorescence was assessed by excitation at 355 nm (emission 450 nm). The geometric mean of the 355 nm fluorescence intensity distribution from each cell population was calculated and normalised to that of the zero glucose control measured on the same day.

### Small interfering RNA (siRNA) knockdown

GLUTag cells were transfected with 50 nmol/l scrambled or *Sglt1* siRNA (Qiagen, Crawley, UK) for 24 h using Lipofectamine 2000. Cells were reseeded into 24-well plates and used 24 h later. Knockdown efficiency was assessed using Taqman assays for *Slc5a1* (sglt1) and *Actb* (β-actin) (Applied Biosystems, Paisley, UK) [7, 21].

### GLP-1 secretion

Secretion studies on GLUTag and primary intestinal cultures were performed 24–36 h after plating in Matrigel-coated 24-well plates. Cultures were incubated with test reagents in saline buffer containing 0.1% fatty acid-free BSA for 2 h at 37°C. Cell lysates were then collected from primary cultures as described previously [7]. Supernatant fractions and lysates were assayed using either a GLP-1-active ELISA kit (Millipore, Watford, UK) or a total GLP-1 assay (MesoScale Discovery, Gaithersburg, MD, USA). For primary cells, GLP-1 secretion was expressed as a fraction of the total hormone content per well, normalised to basal secretion measured in parallel. For GLUTag cells, supernatant fraction concentrations were normalised to basal levels in parallel control wells.

### Calcium imaging

Experiments were performed on 5- to 8-day-old cultures from *Sglt1* ^*−/−*^ mice and wild-type littermates, crossed into the GLU-Venus background. Cells were loaded in 7 μmol/l fura2-AM (Invitrogen, UK) and 0.01% pluronic F127, and incubated in saline buffer containing 1 mmol/l glucose and 300 μmol/l eserine, for 30 min. Experiments were performed using the Olympus IX71 imaging system described above. Fura2 was excited at 340 and 380 nm (emission 510/80 nm), and Venus at 475 nm (emission 535/50 nm). Fura2 fluorescence measurements were taken every 2 s, background corrected, and expressed as the 340/380 nm ratio. Mean fluorescence ratios were determined over 20 s, and responses expressed as the maximum ratio achieved during stimulation divided by the mean of the ratios measured before and after washout. Cells were included in the analysis if they responded to the positive controls, 10 mmol/l glutamine and 30 mmol/l KCl.

### Solutions

Saline buffer contained (mmol/l) 4.5 KCl, 138 NaCl, 4.2 NaHCO_3_, 1.2 NaH_2_PO_4_, 2.6 CaCl_2_, 1.2 MgCl_2_ and 10 HEPES (pH 7.4, NaOH). In some experiments, KCl was increased with equivalent reduction in NaCl, in others Na^+^ was replaced by *N*-methyl-d-glucamine (NMDG). Where possible, solutions were prepared as ×1,000 stock. Glucokinase activator (GKA50) [22] (AstraZeneca, Macclesfield, UK) was dissolved in DMSO. Chemicals were supplied by Sigma Aldrich (Poole, UK) unless otherwise stated.

### Data analysis

Data are presented as means ± SEM. Significance was assessed using Student’s t test (Microsoft Excel) or by one- or two-way ANOVA followed by Bonferroni or Dunnett’s post hoc tests (Graph Pad Prism Software, San Diego, CA, USA). *p* < 0.05 was considered significant.

## Results

### Glucose uptake pathways in L cells

**Fig. 1:**
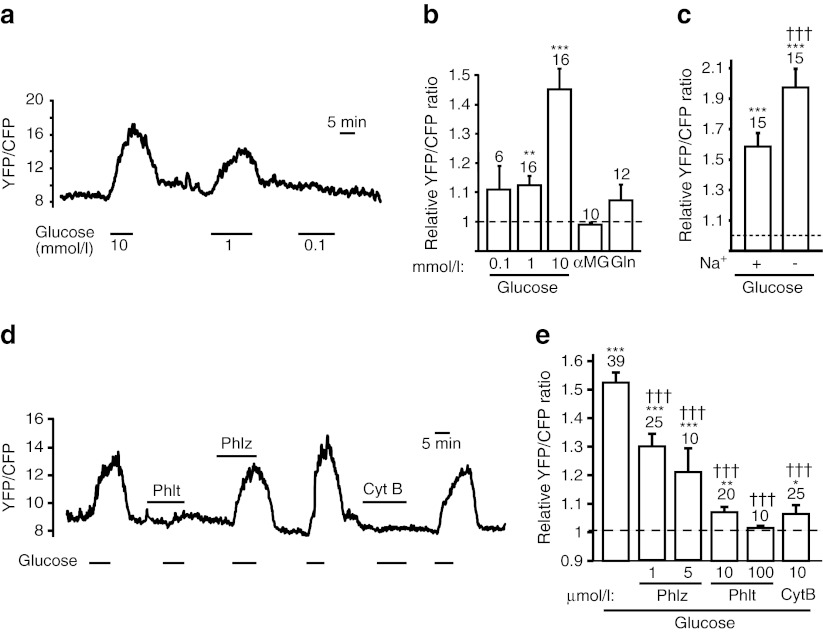
Glucose uptake into GLUTag cells. (**a**) Representative trace showing the raw FRET YFP/CFP ratio monitored in a single GLUTag cell producing FLII^12^Pglu-700μδ6. Glucose (10, 1, 0.1 mmol/l) was applied as indicated by the horizontal bars. (**b**) Mean normalised YFP/CFP ratio, recorded as in (**a**). Glucose (0.1, 1 or 10 mmol/l, applied in random order), αMG (10 mmol/l) and glutamine (Gln, 10 mmol/l) were applied as indicated. Error bars represent 1 SEM from *n* = 6–16 cells as indicated above the bars from two to four separate experiments. (**c**) Mean normalised YFP/CFP ratio, recorded as in (**a**), elicited by 10 mmol/l glucose applied in saline buffer (Na^+^ +) or after Na^+^ substitution by NMDG^+^ (Na^+^ −) in *n* = 15 cells as indicated above the bars. (**d**) Representative trace, recorded as in (**a**). Glucose (10 mmol/l), phloretin (Phlt, 100 μmol/l), phlorizdin (Phlz, 1 μmol/l) or cytochalasin B (Cyt B, 10 μmol/l) was applied as indicated. (**e**) Mean normalised YFP/CFP responses, recorded as in (**d**), to glucose (10 mmol/l), phloridzin (Phlz, 1, 5 μmol/l), phloretin (Phlt, 10, 100 μmol/l) or cytochalasin (CytB, 10 μmol/l) in *n* = 20–29 cells as indicated above the bars from five to seven separate experiments. Statistical significance was tested by ANOVA followed by a Student’s *t* test to compare responses to baseline (*p < 0.05, **p < 0.01, ***p < 0.001) or by Dunnett’s test to compare responses to glucose (^†††^ *p* < 0.001)

Intracellular glucose levels were monitored in GLUTag cells transiently producing the FLII_12_Pglu-700μδ6 glucose FRET probe [18]. Initial experiments confirmed the specificity of the sensor, as glucose (1 or 10 mmol/l), but not glutamine (10 mmol/l) or the glucose analogue αMG (10 mmol/l), elicited a significant increase in the YFP/CFP ratio (Fig. 1a,b). To determine whether glucose entry into L cells is primarily mediated by Na^+^-coupled (SGLT) or facilitative (GLUT) glucose transport, we examined the effects of extracellular Na^+^ replacement and pharmacological inhibitors of both classes of transporter (Fig. 1c–e). The response to 10 mmol/l glucose was impaired in the presence of 1 or 5 μmol/l phloridzin, but appeared increased rather than decreased in the absence of Na^+^. Although the increased glucose signal following Na^+^ substitution could reflect a paradoxical increased net glucose influx, it might also be caused by a reduced rate of glucose metabolism or an altered intracellular ionic composition/pH affecting the properties of the glucose sensor. A robust inhibition of glucose uptake was observed in the presence of either phloretin (10 or 100 μmol/l) or cytochalasin B (10 μmol/l), both of which target the GLUT family.

**Fig. 2:**
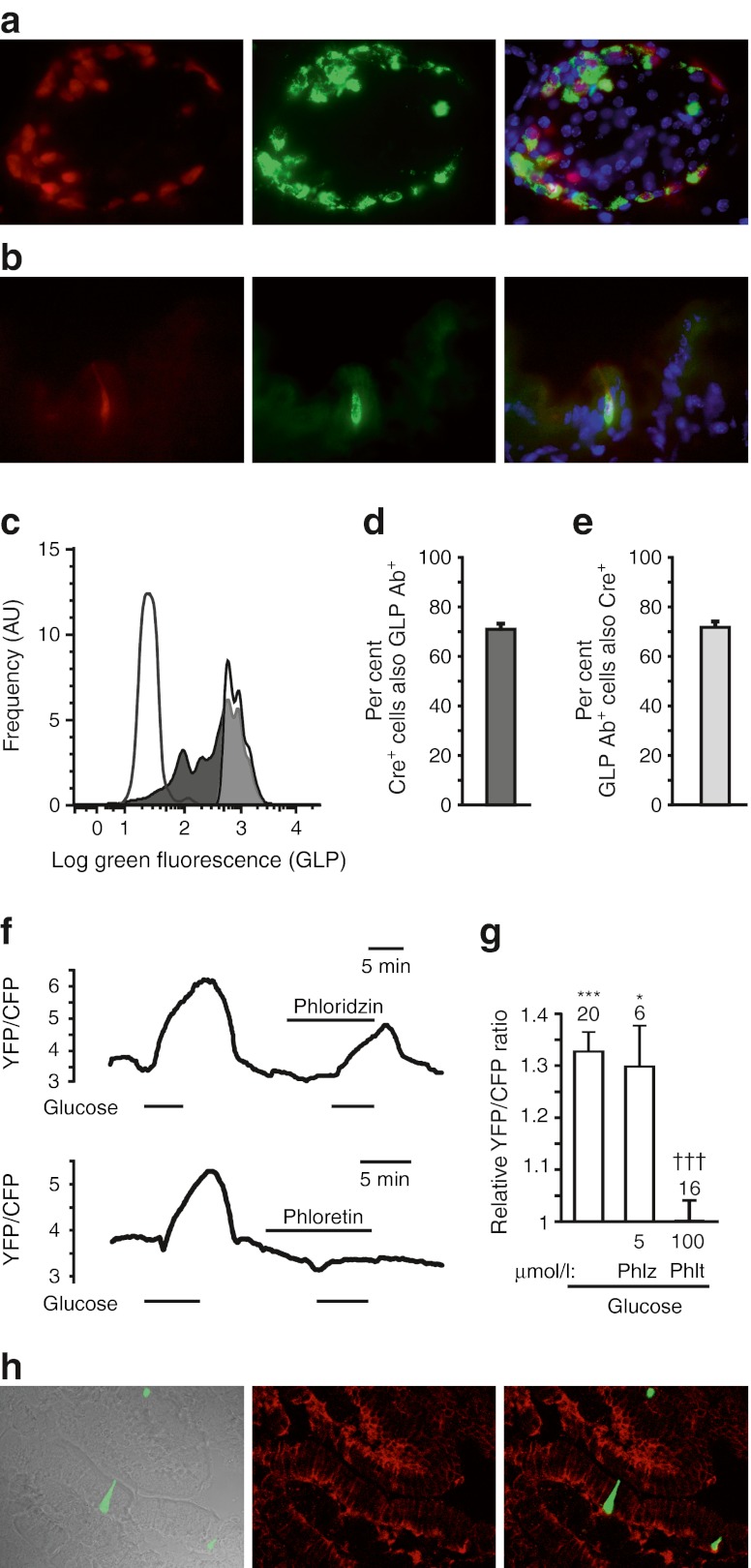
Mechanisms of glucose uptake in primary L cells. (**a**,**b**) Colocalisation of direct tdRFP fluorescence (red) with glucagon immunofluorescence (green) in (**a**) pancreatic and (**b**) colonic tissue slices from a GLU-Cre12×tdRFP mouse. Blue represents Hoechst fluorescence. (**c**) FACS analysis performed on colonic digests from GLU-Cre12×tdRFP mice stained with an antibody against proglucagon and a green fluorescent secondary antibody. Frequency histograms represent the green fluorescence of all tdRFP-positive cells (dark grey shading) or all strongly green fluorescent cells (tdRFP-positive and -negative cells; light grey shading). The non-shaded distribution represents the green fluorescence of tdRFP-positive cells when primary proglucagon antibody was omitted. Frequencies are given in arbitrary units (AU). (**d**,**e**) FACS-determined percentages of tdRFP-producing cells staining for proglucagon (**d**), and of proglucagon-positive cells that contain tdRFP from three independent experiments. (**f**) Intracellular glucose, monitored as YFP/CFP ratio, in individual primary L cells identified by tdRFP fluorescence. Glucose (10 mmol/l), phloridzin (5 μmol/l) or phloretin (100 μmol/l) was applied as indicated. (**g**) Mean normalised FRET responses, recorded as in (**f**) from *n* = 6–20 cells (as indicated above the bars) from more than five separate experiments. *p < 0.05, ***p < 0.001 vs baseline by single-factor *t* tests. Statistical comparison between glucose and other conditions was assessed by ANOVA and Dunnett’s test; ^†††^ *p* < 0.001. (**h**) Immunofluorescent localisation of GLUT2 in a duodenal slice. Left, Phase-contrast image of a villus, with superimposed Venus fluorescence (green) identifying an L cell; middle, GLUT2 immunofluorescence (red); right, overlay of Venus fluorescence and GLUT2 immunofluorescence

As we previously reported an ∼10-fold higher expression of *Sglt1* mRNA in primary L cells compared with GLUTag cells [7], we aimed to perform similar experiments in L cells in primary culture. To enable the use of YFP/CFP-based FRET sensors, we developed a mouse model in which L cells are identifiable by their red fluorescence. Transgenic mice were generated in which *Cre-*recombinase expression is driven by the proglucagon promoter (GLU-Cre12). Crossing these with Rosa26tdRFP reporter mice [15] resulted in red fluorescence in glucagon-positive pancreatic alpha cells (Fig. 2a) and GLP-1-containing L cells (Fig. 2b), as expected. The efficiency of L cell targeting was quantified by FACS analysis of colonic epithelial cell suspensions from GLU-Cre12×tdRFP mice, revealing that >70% of proglucagon-positive cells had undergone Cre-mediated recombination (Fig. 2c–e). We detected a fraction of cells (<30%) with red fluorescence not staining for proglucagon, corresponding to a small number of red fluorescent cells in primary colonic cultures not showing the typical morphology of L cells. These were excluded morphologically from experiments on mixed epithelial primary cultures transduced with a newly made adenovirus expressing *FLII*_*12*_ *Pglu-700μδ6*. In L cells in primary culture, 10 mmol/l glucose elicited an average 1.34-fold increase in the YFP/CFP ratio, which was abolished by phloretin (100 μmol/l) but unaffected by phloridzin (5 μmol/l) (Fig. 2f,g). The glucose transporter GLUT2 was detected in the basolateral membrane of enterocytes and L cells (Fig. 2h).

### Glucose-stimulated changes in L cell metabolism

**Fig. 3:**
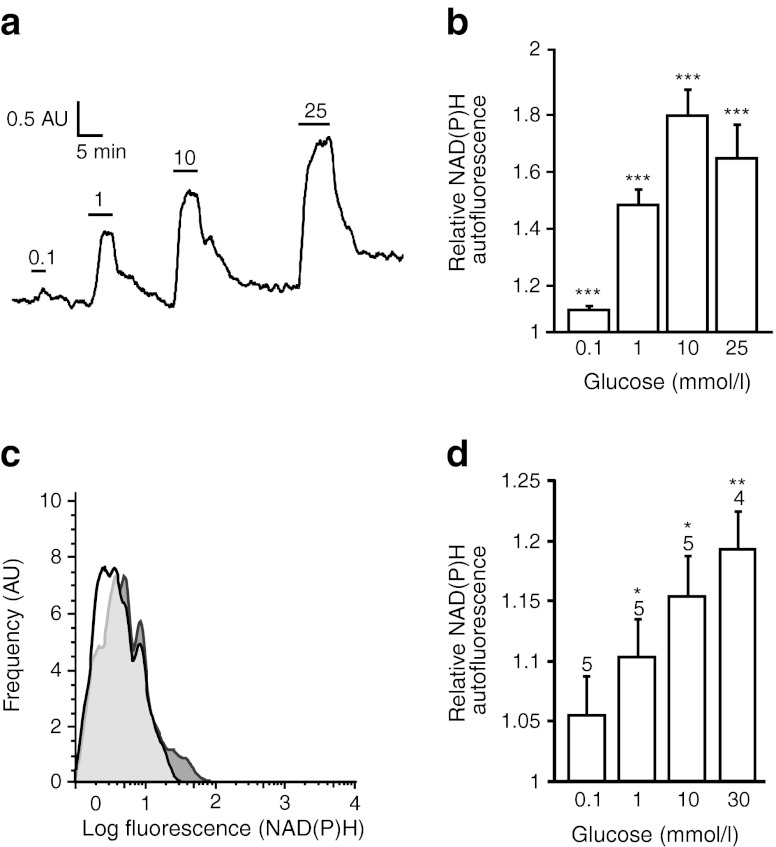
Glucose-dependent changes in L cell metabolism. (**a**) Representative trace representing NAD(P)/NAD(P)H autofluorescence (arbitrary units, AU) from a GLUTag cell upon glucose application (0.1, 1, 10, 25 mmol/l). (**b**) Mean normalised autofluorescence, recorded as in (**a**). *n* = 81 cells from five separate experiments. (**c**) Smoothed frequency histograms showing the autofluorescence intensity of primary L cells in small intestinal digests in the absence (white-shaded) and presence (dark grey-shaded) of 10 mmol/l glucose. For each condition, 1 million events were counted, of which ∼0.1% were Venus positive. (**d**) Normalised mean autofluorescence intensities of primary L cells, analysed as in (**c**), from four to five mice analysed independently as indicated above the bars. *p < 0.05, **p < 0.01, ***p < 0.001 vs baseline, by Student’s single-sample *t* test

To investigate whether elevation of intracellular glucose translates into metabolic changes within GLUTag cells, we monitored the autofluorescence signal attributed to NAD(P)H by real-time imaging (Fig. 3a). Glucose dose-dependently increased NAD(P)H autofluorescence, eliciting a maximal 1.8-fold increase at 10 mmol/l (Fig. 3b). To monitor NAD(P)H in primary L cells, we developed a FACS analysis-based technique for use with acutely dispersed SI epithelial cells. In the Venus-labelled L cell subpopulation, glucose dose-dependently shifted the histogram of NAD(P)H autofluorescence rightwards (Fig. 3 c,d).

### Role of glucokinase in L cells

**Fig. 4:**
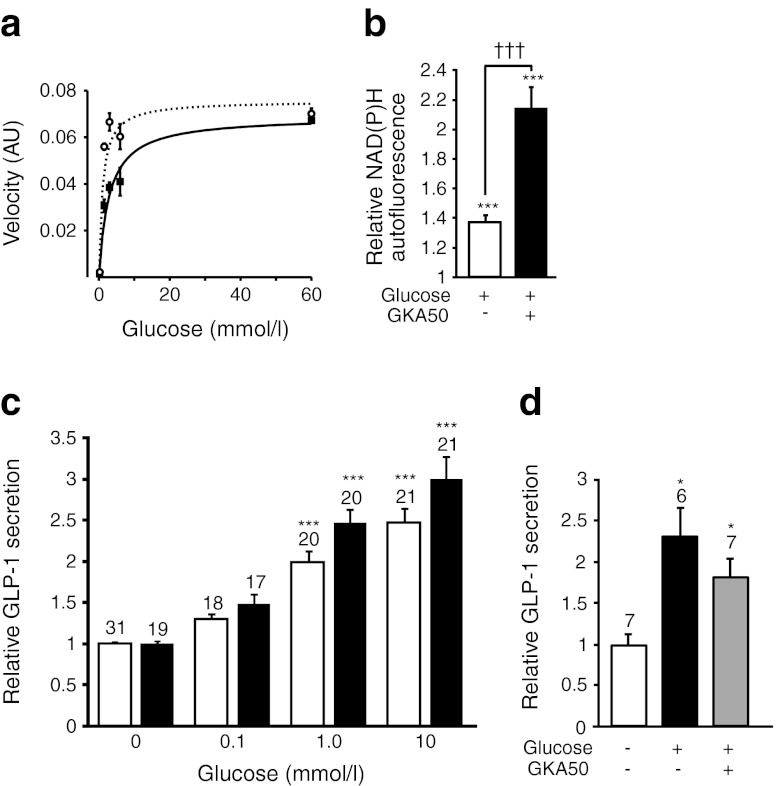
Role of glucokinase in GLUTag and primary L cells. (**a**) Glucokinase activity in GLUTag extracts vs glucose concentration in the absence (black squares, solid line) and presence (white circles, dotted line) of GKA50 (3 μmol/l). Velocity represents the increase in fluorescence over time in arbitrary units (AU). Data were fitted using a hyperbola plot with the equation y=*v*_max_[glucose]/(S_0.5_ + [glucose]), giving values of 3 and 1 mmol/l for *S*_0.5_, the substrate concentration at which half the maximal velocity (*V*_max_) is reached, in the absence and presence of GKA50, and *V*_max_ under both conditions of 0.07 AU (*n* = 4). (**b**) Mean normalised NAD(P)H autofluorescence responses in GLUTag cells after addition of glucose (3 mmol/l) and GKA50 (3 μmol/l). *n* = 54 cells from three separate experiments. Statistical comparison to autofluorescence in the absence of glucose (***p < 0.001) and between the absence/presence of GKA50 (^†††^ *p* < 0.001) were assessed by Student’s *t* test. (**c**) GLP-1 secretion from GLUTag cells in response to various concentrations of glucose, with GKA50 (3 μmol/l, black bars) or without GKA50 (white bars). Secretion was normalised to parallel baseline measurements and the number of wells is shown above the bars. Statistical comparisons were assessed by two-way ANOVA (*p* = 0.0047 for GKA50 vs no GKA50, and *p* < 0.0001 for effect of glucose concentrations, with no significant interaction), followed by post hoc Bonferroni’s test; ***p < 0.001 vs respective controls in the absence or presence of GKA50. (**d**) GLP-1 secretion from primary upper SI cultures in response to 1 mmol/l glucose with or without GKA50 (3 μmol/l). Secretion was calculated relative to GLP-1 content, normalised to basal secretion measured in parallel, with the number of wells given above the bars. Significance was assessed by one-sample *t* test; *p < 0.05 vs control

To examine the role of the gene encoding glucokinase (*Gck*), which is expressed in GLUTag and L cells at mRNA levels comparable to those found in pancreatic beta cells [7], we measured enzyme activity in GLUTag cell extracts [20]. Consistent with functional glucokinase production, the enzymatic rate increased with increasing glucose concentrations in the mmol/l range, at which low-affinity hexokinases would be saturated, and a significant (*p* < 0.001) further increase was observed when a glucokinase activator, GKA50 (3 μmol/l), was added to the cell extract (Fig. 4a). Addition of GKA50 to 3 mmol/l glucose also significantly increased the NAD(P)H autofluorescence in GLUTag cells (Fig. 4b). In secretion experiments, GKA50 caused a small but significant increase in glucose-stimulated GLP-1 release from GLUTag cells (*p* < 0.01 by two-way ANOVA, Fig. 4c). However, under the conditions tested, we were unable to detect an effect of GKA50 on GLP-1 secretion from primary intestinal cultures (Fig. 4d).

### Effect of intracellular glucose on GLP-1 secretion

**Fig. 5:**
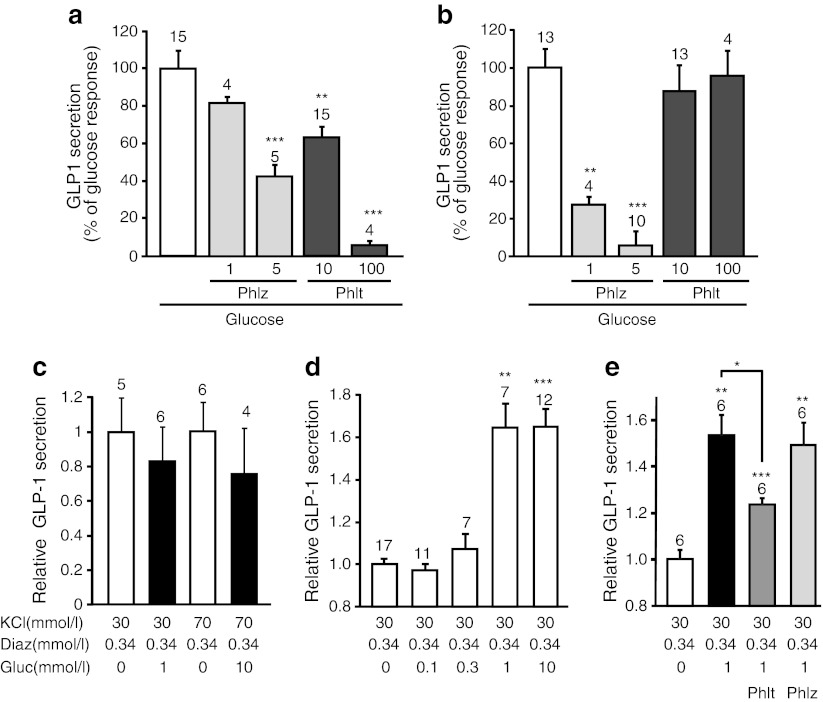
Pharmacological modulation of glucose-stimulated GLP-1 secretion. GLP-1 secretion from (**a**) GLUTag cells and (**b**) primary upper SI cultures, in glucose (1 mmol/l), phloretin (Phlt, 10 or 100 μmol/l) and phloridzin (Phlz, 1 or 5 μmol/l), as indicated. Incremental responses to glucose are shown for each condition, normalised to the incremental glucose response in the absence of inhibitor (100%). Error bars represent 1 SEM and the number of wells is given above the bars. Significance was assessed by one-way ANOVA and Dunnett’s test vs glucose only; **p < 0.01, ***p < 0.001. (**c**) GLP-1 secretion from primary upper SI cultures in 30 or 70 mmol/l extracellular K^+^ and 340 μmol/l diazoxide (Diaz) in the absence (white bars) or presence (black bars) of glucose (1 or 10 mmol/l) as indicated. Error bars represent 1 SEM and the number of wells is given above the bars. Secretion was calculated relative to GLP-1 content and normalised to basal secretion measured in parallel. (**d**,**e**) GLP-1 secretion from GLUTag cells in 30 mmol/l extracellular K^+^ and 340 μmol/l diazoxide and (**d**) various glucose concentrations or (**e**) 1 mmol/l glucose with or without phloretin (10 μmol/l, Phlt) or phloridzin (5 μmol/l, Phlz) as indicated. Secretion was normalised to baseline secretion measured in parallel. Error bars represent 1 SEM and the number of wells is given above the bars. Significance was determined by one-way ANOVA followed by one-sample *t* tests and Dunnett’s test vs glucose in the absence of inhibitor; *p < 0.05, **p < 0.01, ***p < 0.001

To evaluate whether glucose-dependent GLP-1 release requires activation of an intracellular target, such as a signal generated from glucose metabolism, we examined whether GLUT inhibition, which largely abolished intracellular glucose transients, affected GLP-1 secretion. In primary intestinal cultures, the secretory response to 1 mmol/l glucose was not significantly impaired by phloretin (10 or 100 μmol/l), whereas the higher dose abolished secretion from GLUTag cells (Fig. 5a,b). In contrast, in both model systems, glucose-triggered secretion was significantly reduced when SGLTs were inhibited by phloridzin, with more profound effects evident in the primary cultures (Fig. 5 a,b). Consistent with a dominant role of electrogenic Na^+^-coupled glucose uptake in primary L cells, no further glucose-dependent increase in GLP-1 secretion was observed in primary cultures depolarised by KCl (Fig. 5c). Glucose-dependent amplification of secretion was, however, seen under these conditions in GLUTag cells (Fig. 5d), which was sensitive to phloretin but not phloridzin (Fig. 5e).

### Role of SGLT1 in glucose-triggered GLP-1 secretion

**Fig. 6:**
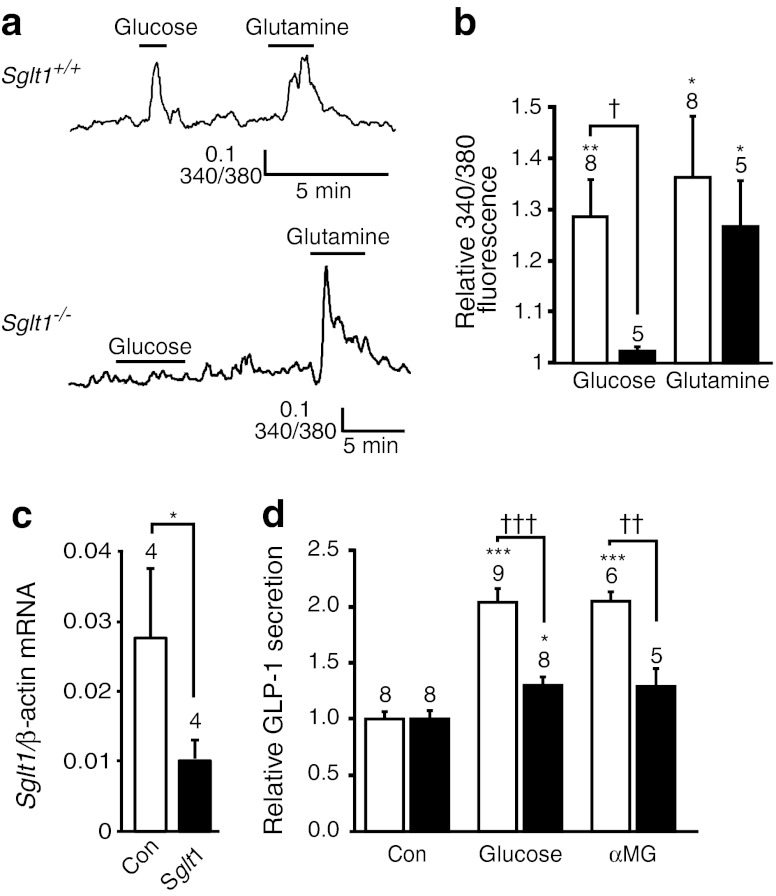
Effect of genetic interference with SGLT1 action. (**a**) Ca^2+^ concentrations in Venus-positive L cells in colonic cultures, monitored as the fura2 340/380 nm fluorescence ratio. Traces from a wild-type and an *Sglt1* ^*−/−*^ mouse are shown after addition of glucose (10 mmol/l) and glutamine (10 mmol/l). The vertical bar represents a change in the fluorescence ratio of 0.1. (**b**) Mean normalised Ca^2+^ responses in L cells recorded as in (**a**) from colonic cultures of control (white bars) and *Sglt1* ^*−/−*^ (black bars) mice. Error bars represent 1 SEM and the number of cells is given above the bars. *p < 0.05, **p < 0.01 vs baseline and ^†^ *p* < 0.05 between genotypes assessed using Student’s *t* test. (**c**) *Sglt1* expression in GLUTag cells transfected with *Sglt1* or scrambled (Con) siRNA, as determined using quantitative RT-PCR, and normalised to *β-actin*. mRNA from four experiments were analysed for each bar. Data are presented as geometric mean and upper SEM calculated from the log (base 2) data. Significance was analysed by Student’s *t* test on the non-transformed ΔC_t_ data; *p < 0.05. (**d**) GLP-1 secretion from GLUTag cells transfected with scrambled (white bars) or *Sglt1* siRNA (black bars). Cells were incubated in the absence of additions (Con) or in the presence of glucose (10 mmol/l) or αMG (100 mmol/l). Secretion was normalised to baseline measured in parallel on the same day. Error bars represent 1 SEM, and the number of wells tested for each concentration is indicated above the bar. Significance was calculated by two-way ANOVA and Bonferroni test; *p < 0.05, ***p < 0.001 vs baseline; ^††^ *p* < 0.01, ^†††^ *p* < 0.001 between knock-down and control

To further confirm the role of SGLT1 in glucose-stimulated GLP-1 secretion, we crossed GLU-Venus mice with the recently described *Sglt1* knockout mouse model [14]. Glucose (10 mmol/l) triggered a rise in cytosolic Ca^2+^ in identified L cells in colonic cultures from *Sglt1* ^*+/+*^ but not *Sglt1* ^*−/−*^ mice (Fig. 6a,b). Glutamine, in contrast, stimulated similar Ca^2+^ responses in L cells from both wild-type and knockout tissue cultures. This is consistent with the previous observation that *Sglt1* knockout selectively abolishes glucose-stimulated GLP-1 secretion [14].

As the impaired Ca^2+^ response and GLP-1 secretion of L cells in this SGLT1-deficient model may still arise from a defect in glucose uptake into neighbouring cells rather than the enteroendocrine cells themselves, we also knocked down *Sglt1* in GLUTag cells using siRNA, which decreased mRNA expression by ∼60% (Fig. 6c). *Sglt1* knockdown largely abolished the effects of both glucose (10 mmol/l) and αMG (100 mmol/l) on GLP-1 release (Fig. 6d).

## Discussion

The mechanism underlying glucose sensing by L cells is a topic of recent debate, with sweet taste receptors (Tas1R2/3) [23, 24], SGLT1 [12] and K_ATP_ channels [16] each suggested to play a role. We demonstrate here that genetic or pharmacological interference with SGLT1 abolishes glucose-triggered Ca^2+^ responses and GLP-1 secretion from L cells in primary culture. This is consistent with the inhibition of GLP-1 secretion by phloridzin from perfused intestinal preparations [10] and the more recent observation of impaired glucose-triggered GLP-1 secretion in *Sglt1* knockout mice [14]. The latter observations are incompatible with the concept of an apically located glucose receptor on the surface of the L cell, as inhibition of glucose absorption would, if anything, tend to increase exposure of such a receptor to luminal glucose. Although they could be explained by a basolaterally expressed receptor which is exposed to elevated glucose concentrations after SGLT1-dependent absorption through enterocytes, the current findings, in which both sides of the cells are exposed to glucose, argue against any major role of an extracellular receptor. This is consistent with our previous observation that artificial sweeteners, at concentrations that saturate Tas1R2/3 receptors, did not trigger GLP-1 release from primary intestinal cultures [7]. A metabolic sensing mechanism downstream of SGLT1-mediated glucose uptake could also be envisioned. Monitoring intracellular glucose levels, however, allowed us to dissociate the stimulatory action of Na^+^-coupled glucose uptake from possible downstream metabolic effects, as SGLT inhibition had only minor effects on intracellular glucose levels, whereas GLUT inhibitors largely abolished glucose uptake in L cells but did not significantly impair GLP-1 secretion. The finding that *Sglt1* knockdown in GLUTag cells impaired both glucose- and αMG-triggered secretion argues for a mechanism intrinsic to the enteroendocrine cells, rather than involving coupling through neighbouring enterocytes. It also demonstrates a dominant role of SGLT1 over SGLT3, which is also produced in GLUTag and primary L cells [7]. We thus conclude that the electrogenic uptake itself via apically localised SGLT1 [7] is the major glucose-sensing mechanism in L cells.

To enable the use in primary tissues of FRET-based sensors employing YFP and CFP, we generated a new BAC transgenic mouse model expressing the gene encoding *Cre* recombinase under control of the proglucagon promoter, targeting enteroendocrine L cells as well as pancreatic alpha cells unlike the shorter promoter constructs used previously to drive *Cre* expression in alpha cells [25]. Quantification by FACS analysis revealed that, although the majority of proglucagon-stained cells in the colon also contained the Cre reporter in GLU-Cre12×tdRFP mice, ∼30% did not. This suggests that a small but significant number of L cells may escape Cre recombination, and should be taken into account when GLU-Cre12 mice are used in future conditional gene knockout experiments. Cre-mediated activation of RFP production was also evident in some cells that did not stain for proglucagon, consistent with the observation that a small proportion of the red fluorescent cells in primary colonic cultures did not exhibit morphology typical of L cells. These cells were readily identifiable and could be avoided in single-cell imaging experiments, but are likely to reflect transient transgene activation in a different cell population during development. Similar findings in the other founder GLU-Cre strains (see ESM Table 2) suggest that this is not merely an artefact of the particular transgene integration site.

Previous analysis detected *Glut1*, *Glut2* and *Glut5* expression in primary murine L cells, with *Glut2* evident in L cells from the SI, and *Glut1* in those from the colon [7]. GLUTag cells notably lack *Glut2* [12], but express *Glut3*, as determined by Affymetrix microarrays (data not shown). The role of GLUTs in incretin hormone secretion is unclear, as their pharmacological inhibition had no significant effect on glucose-stimulated GLP-1 secretion in primary cultures, although mice lacking GLUT2 showed reduced plasma GLP-1 concentrations following oral glucose, and a lower intestinal GLP-1 content [26]. Whereas SGLT1 is apically located on L cells [14], GLUT2 appeared localised to the basolateral surface of L cells and enterocytes, suggesting that intracellular glucose concentrations would reflect basolateral rather than luminal glucose levels. Whereas GLP-1 secretion is predominantly stimulated by oral rather than systemic glucose delivery, GLP-1 release from the perfused pig intestine was found to be influenced also by vascular glucose levels [27], possibly through alteration of the intracellular glucose concentration in L cells.

Whether intracellular glucose metabolism plays any role in determining GLP-1 secretion remains uncertain. In GLUTag but not primary L cells, we observed a strong inhibition of secretion when glucose uptake was completely blocked and an amplifying action of glucose under depolarising conditions. The balance between the metabolic and electrogenic effects of glucose is thus slightly different between the cell line and primary culture, with a more dominant role for SGLT1-based glucose sensing in the latter. NAD(P)H autofluorescence measurements suggest that GLUTag and primary L cells increase their metabolic rate in response to extracellular glucose elevation, consistent with our previous observation that ATP concentrations in GLUTag cells are elevated upon exposure to 1 mmol/l glucose [28]. NAD(P)H changes occurring at glucose concentrations above ∼1 mmol/l would be consistent with the recruitment of *Glucokinase*, which is known to be expressed in enteroendocrine cells [7, 9, 29–31]. Glucokinase activity was demonstrable in GLUTag cell extracts, and was responsive to the glucokinase activator, GKA50. The observed *S*_0.5_ value (∼3 mmol/l) in the absence of GKA50 is lower than the expected value of ∼5-8 mmol/l [20], possibly reflecting incomplete inhibition of hexokinases I–III or additional regulation of enzyme activity by unknown factors in GLUTag cell extracts. GKA50 significantly affected NAD(P)H autofluorescence at 3 mmol/l glucose in GLUTag cells, demonstrating that glucokinase exhibits at least some control over the metabolic flux in L cells, but had only a small effect on glucose-stimulated GLP-1 secretion from GLUTag cells and no effect on secretion from primary cultures.

The present study demonstrates that metabolism plays at best a minor role in glucose-stimulated GLP-1 secretion in primary cultures, consistent with the finding that non-metabolisable glucose analogues such as αMG are effective stimuli of GLP-1 release in vivo and in vitro [10, 11, 16, 32]. Although phloretin abolished glucose accumulation in GLUTag and primary L cells, its effect on glucose-stimulated GLP-1 secretion was restricted to the cell line, suggesting that glucose metabolism does not enhance secretion in the context of a predominant SGLT1-mediated stimulus. Future work should address whether the glucokinase/K_ATP_ channel machinery exerts longer-term effects on L cells or enables modulation of GLP-1 secretion by neurohormonal or alternative nutritional stimuli.

## Electronic supplementary material

Below is the link to the electronic supplementary material.

ESM Methods PDF 95 kb

ESM Table 1 PDF 39 kb

ESM Table 2 PDF 13 kb

## Abbreviations

BAC: Bacterial artificial chromosome
CFP: Cyan fluorescent protein
CMV: Cytomegalovirus
FRET: Förster resonance energy transfer
GIP: Glucose-dependent insulinotropic polypeptide
GKA: Glucokinase activator
GLP-1: Glucagon-like peptide-1
K_ATP_: ATP-sensitive potassium channel
αMG: α-Methyl-d-glucopyranoside
NMDG: *N*-Methyl-d-glucamine
RFP: Red fluorescent protein
SGLT1: Sodium-dependent glucose transporter 1
SI: Small intestine
tdRFP: Tandem red fluorescent protein
YFP: Yellow fluorescent protein
