# Supplementary material for: Predominant role of active versus facilitative glucose transport for glucagon-like peptide-1 secretion
Source: Diabetologia. 2012 May 26;55(9):2445–55. doi: 10.1007/s00125-012-2585-2 (PMC3411305; doi:10.1007/s00125-012-2585-2)
Supplement: Supplementary file 1 — PDF 95 kb [file 125_2012_2585_MOESM1_ESM.pdf]

ESM Methods: Creation of proglucagon-promoter driven Cre-recombinase expressing transgenic mice

To express *Cre*-recombinase *iCre* (a kind gift by Rolf Sprengel, Max Planck Institute for Medical Research, Heidelberg, Germany) under the control of the proglucagon promoter we replaced the sequence between the proglucagon start codon in exon 2 and stop codon in exon 6 in the murine based bacterial artificial chromosome (BAC) RP23-343C17 (Children's Hospital Oakland Research Institute, Oakland, CA, USA) by the *iCre* sequence using Red/ET recombination technology (Genebridges, Heidelberg, Germany). Briefly, *iCre* sequence was amplified by PCR adding proglucagon gene specific 3' and 5' sequences (see oligonucleotides tabulated below) and homologous recombination was achieved upon co-transforming an rpsLneo-modified BAC (Reimann et al. 2008) containing E.coli DH10B clone with the PCR product and the plasmid pSC101-BAD-gbaA, which provides the recombination enzymes (Genebridges). Positive recombinants were isolated using appropriate antibiotic selection and characterised by PCR and restriction analysis. Identity and correct positioning of the introduced *iCre* sequence was confirmed by direct sequencing. BAC-DNA for microinjection was purified using the large-construct Maxi-Prep kit (Qiagen, Crawley, UK) and dissolved at ~ 1-2 ng/μl in injection buffer containing (mmol/l): 10 Tris-HCl pH 7.5, 0.1 EDTA, 100 NaCl, 0.03 spermine, 0.07 spermidine. Pronuclear injection into ova derived from C57B6/CBA F1 parents and re-implantation of embryos into pseudo-pregnant females was performed by the Central Biomedical Services at Cambridge University. DNA of pups was isolated from ear clips by proteinase K digestion and screened for the transgene by PCR using the following primer pairs: mGlu008/iCre001, iCre002/003, tdRFP-anti/sense (and RM41/42, which amplifies *β-catenin* sequence used as a DNA quality control). Transgene copy

number was estimated by RT-PCR comparing CT numbers for a transgene specific probe (iCre-004, 005 and -probe) and Kir6.2 (Kcnj11-forw, -rev and -probe). The same probes were used with a ΔΔCT method to identify homozygous offspring after back-crossing into C57B6 for at least 7 generations. Initially four transgenic strains were established (estimated transgene copy number): GLU-Cre01 (7-8), Glu-Cre12 (2-3), GLU-Cre14 (1) and GLU-Cre30 (1).
