# Supplementary material for: Predominant role of active versus facilitative glucose transport for glucagon-like peptide-1 secretion
Source: Diabetologia. 2012 May 26;55(9):2445–55. doi: 10.1007/s00125-012-2585-2 (PMC3411305; doi:10.1007/s00125-012-2585-2)
Supplement: Supplementary file 2 — PDF 39 kb [file 125_2012_2585_MOESM2_ESM.pdf]

Supplementary Table 1 (ESM table 1): Oligonucleotides used to create and verify GLU-Cre mice

Name	Sequence
iCre001	GTA GTC CCT CAC ATC CTC AGG
iCre002	GAC AGG CAG GCC TTC TCT GAA
iCre003	CTT CTC CAC ACC AGC TGT GGA
iCre004	GCC GAA ATT GCC AGA ATC AG
iCre005	CAA TGT GGA TCA GCA TTC TCC
iCre-probe	6-FAM-TGA AGG ACA TCT CCC GCA CCG-TAMRA
mGLU008	AAT TGA GCT CAT TTG GAC TGC C
mGLUCre1	TGC TCC CCC ATC ACC CCC TAC CCA CCC CCA TTC TGT GTT CCA TCA GGC AGA AAA AAA ATC CAC CAT GGT GCC CAA GAA GAA GAG G
mGLUCre2	TAC ATC CCA AGT GAC TGG CAC GAG ATG TTG TGA AGA TGG TTG TGA ATG GTG AAA TAC CTA GTC CCC ATC CTC GAG CAG CCT C
RM41	AAG GTA GAG TGA TGA AAG TTG TT
RM41	CAC CAT GTC CTC TGT CTA TTC
mKcnj11-fw	CCC GCT TCG TGT CCA AGA
mKcnj11-rev	CAG CGT GGT GAA CAC ATC CT
mKcnj11-probe	6-FAM-CAA CGT CGC CCA CAA GAA CAT TCG A-BHQ-1
tdRFPsense	CTG TTC CTG GGG CAT GGC
tdRFPanti	CTA CAG GAA CAG GTG GTG G
