# Supplementary material for: Predominant role of active versus facilitative glucose transport for glucagon-like peptide-1 secretion
Source: Diabetologia. 2012 May 26;55(9):2445–55. doi: 10.1007/s00125-012-2585-2 (PMC3411305; doi:10.1007/s00125-012-2585-2)
Supplement: Supplementary file 3 — PDF 13 kb [file 125_2012_2585_MOESM3_ESM.pdf]

Supplementary Table 2 (ESM table 2): Immunhistochemical characterization of GLU-Cre mice derived from different founders:

GluCre Mice	***Strain 01***	***Strain 12***	***Strain 14***	***Strain 30***
Number of tdRFP+ve cells	101	213	69	177
Number of tdRFP+ve cells that express glucagon	27	122	46	116
**% tdRFP+ve cells that are glucagon+ve**	**27**	**57**	**67**	**66**
Number of glucagon+ve cells	102	120	107	105
Number of glucagon+ve cells that are tdRFP+ve	85	82	59	76
**% glucagon+ve cells that are tdRFP+ve**	**83**	**68**	**55**	**72**

Intestinal tissue sections from GLU-Cre mice crossed with Rosa26tdRFP mice (a kind gift by Hans Joerg Fehling, University of Ulm, Germany) were stained for glucagon as described in the main manuscript and the number of cells as given in table 2 were assessed for red and green (glucagon) fluorescence. Further quantification assessing Cre-penetrance and aberrant activation in proglucagon positive and negative cells in the GLU-Cre12 line was performed by FACS analysis as described in the main manuscript.
